# Promising galactose-decorated biodegradable poloxamer 188-PLGA diblock copolymer nanoparticles of resibufogenin for enhancing liver cancer therapy

**DOI:** 10.1080/10717544.2017.1373165

**Published:** 2017-09-12

**Authors:** Hao Dong, Li Tian, Meng Gao, Hong Xu, Chenghong Zhang, Li Lv, Jianbin Zhang, Changyuan Wang, Yan Tian, Xiaochi Ma

**Affiliations:** a College of Pharmacy, Dalian Medical University, Dalian, China;; b Department of Pharmaceutics, The First Affiliated Hospital of Dalian Medical University, Dalian, China;; c College of Basic Medical Sciences, Dalian Medical University, Dalian, China

**Keywords:** Liver cancer, asialoglycoprotein receptor, resibufogenin, Gal-SP188–PLGA, active liver-targeting nanoparticles

## Abstract

Liver cancer is one of the major diseases affecting human health. Modified drug delivery systems through the asialoglycoprotein receptor, which is highly expressed on the surface of hepatocytes, have become a research focus for the treatment of liver cancer. Resibufogenin (RBG) is a popular traditional Chinese medicine and natural anti-cancer drug that was isolated from Chansu, but its cardiotoxicity and hydrophobicity have limited its clinical applications. Galactosyl-succinyl-poloxamer 188 and galactosyl-succinyl-poloxamer 188-polylactide-*co*-glycolide (Gal-SP188–PLGA) were synthesized using galactose, P188, and PLGA to achieve active liver-targeting properties. RBG-loaded Gal-SP188–PLGA nanoparticles (RGPPNs) and coumarin-6-loaded Gal-SP188–PLGA nanoparticles (CGPPNs) were prepared. The *in vitro* cellular uptake, cytotoxicity, and apoptosis of nanoparticles in HepG2 cells were analyzed. The *in vivo* therapeutic effects of nanoparticles were assessed in a hepatocarcinogenic mouse model. The results showed that Gal-SP188–PLGA was successfully synthesized. The cellular uptake assay demonstrated that CGPPNs had superior active liver-targeting properties. The ratio of apoptotic cells was increased in the RGPPN group. In comparison to the other groups, RGPPNs showed superior *in vivo* therapeutic effects and anticancer efficacy. Thus, the active liver-targeting RGPPNs, which can enhance the pharmacological effects and decrease the toxicity of RBG, are expected to become a promising and effective treatment for liver cancer.

## 1. Introduction

Primary liver cancer (PLC) is a common tumor worldwide and the third leading cause of tumor-related death (Cong & Wu, 2015; Rude & Crippin, 2015). The majority of PLC has been diagnosed in developing countries, and hepatitis B virus and aflatoxin were considered as two major causes of liver damage (Zhang et al., 2015). Other causes included dietary and genetic factors. The etiology and pathogenesis of PLC are not yet entirely clear, and the molecular mechanism underlying PLC involves the activation of oncogenes and inactivation of tumor suppressor genes. Current therapies for liver cancer encompass surgical resection, liver transplantation, interventional therapy, and drug treatment. However, although anticancer drugs injure cancer cells directly, they simultaneously damage normal cells, resulting in bone marrow suppression or damage to other organs. New anticancer drug preparations have become a research focus for the treatment of liver cancer and even for other cancers. It is thought that traditional Chinese medicine (TCM) therapy could be a supplementary treatment for liver cancer because of its advantages, which include reducing the toxicities of radiotherapy and chemotherapy, relieving cancer-related symptoms, improving quality of life and extending life span (Liao et al., 2015). However, techniques to improve the targeting ability of the main effective constituents of TCM to the tumor site and to improve the pharmacodynamics of TCM have been a focus of research. In recent years, some studies have confirmed that there are receptors that bind specifically to certain groups (Yan et al., 2015) on the surface of hepatocytes. Asialoglycoprotein receptor (ASGPR), a C-type lectin, is mainly present on the surface of liver parenchymal cells on the side facing the sinusoids. ASGPR is an endocytic receptor that can recognize and bind to oligosaccharides or oligosaccharide proteins specifically containing galactosyl or *N*-acetylgalactosaminyl groups (Ishibashi et al., 1994; Rigopoulou et al., 2012). ASGPR shows high expression on the surface of hepatocytes and hepatocellular carcinoma cell lines (Zhu et al., 2013). The preparation of galactosyl or *N*-acetylgalactosaminyl-modified drug delivery systems, by recognizing ASGPR specifically and increasing the drug concentration in liver cells, can facilitate active targeting to the liver, and consequently, it has gradually become a new treatment option for liver cancer (Cheng et al., 2012; Wang et al., 2012).

According to trends in the development of the treatment of PLC, resibufogenin (RBG) was chosen in this study to treat PLC. RBG (3-hydroxy-14,15-epoxy-20,22-diennolide glycoside, C_24_H_32_O_4_) is an effective component of toad venom that belongs to steroid compounds and its chemical structure is shown in the Supplementary data. It is mainly obtained from the skin and parotid venom glands of toads, including *Bufo bufo gargarizans* Cantor and *Bufo melanostictus* Schneider (Zhang et al., 2013). RBG has extensive physiological and pharmacological functions, such as cardiotonic effects, platelet inhibition, vascular contraction, and antiepileptic and local anesthetic actions (Xie et al., 2001; Wang et al., 2014). However, high dosages of RBG have also demonstrated cardiac toxicity, and the cardiotoxicity mechanisms were the inhibition of membrane depolarization and Na^+^, K^+^-ATPase activity (Xie et al., 2001; Bick et al., 2002). Previous studies have also suggested that RBG, as an important active ingredient of toad venom, shows significant anticancer activity both *in vitro* and *in vivo*. RBG can inhibit the growth of a variety of cancer cells, inhibit cell proliferation, induce cell differentiation, induce cancer cell apoptosis, disrupt cell cycle, inhibit cancer angiogenesis, reverse multi-drug resistance, and regulate the immune response, and present a concentration-dependent and time-dependent effect (Huang et al., 2006; Meng et al., 2006; Wang et al., 2006; Xiao et al., 2006; Qi et al., 2011; Qiu et al., 2013). However, the poor solubility and strong cardiotoxicity of RBG limit its anticancer activity in clinical applications. Therefore, the development of a new dosage form to increase the solubility and improve the targeting of RBG to reduce its cardiotoxicity and side effects has become a recent research focus.

Polylactide-*co*-glycolide (PLGA) has been approved by the Food and Drug Administration (FDA) as one kind of pharmaceutical excipient for injection (Mundargi et al., 2008). PLGA has some advantages, such as good biocompatibility and biodegradable and non-immunogenic properties. Thus, PLGA is often used as drug carrier to prepare certain dosages. In recent years, research has also focused on modified PLGA copolymers, such as the use of PEG-modified PLGA nanoparticles (NPs), to increase the drug circulation time (Avgoustakis et al., 2003). Poloxamer 188 (P188), which has also been approved by the FDA, is a type of three-block copolymer consisting of poly(ethylene oxide) (PEO) and hydrophobic poly(propylene oxide) (PPO) units, and the subtypes of poloxamer were determined by different numbers of PEO and PPO units. Some studies have shown that P188-modified nanomaterials could increase the cytotoxic activity of the anticancer drug by 2–3 orders of magnitude in drug-resistant tumor cells (Kabanov et al., 2002). The mechanism by which P188 reverses drug resistance occurs through an influence on the expression of adenosine triphosphate (ATP) binding cassette (ABC) transporters, and thus it can increase intracellular drug accumulation and strengthen drug efficacy (Batrakova et al., 2001). P188 is commonly used pharmaceutically to emulsify or improve drug solubility because it is soluble in both water and organic solvent (Zhang et al., 2010).

To improve the bioavailability and decrease the cardiotoxicity of RBG, NPs were chosen as the drug delivery system for RBG in this study. NPs, as targeting preparations, have features such as low toxicity, sustained release, and good stability. By controlling the particle size and surface charge, NPs exhibit a passive targeting effect, and by modifying the surface of the carrier material, NPs escape identification by the mononuclear phagocytic system. By linkage to a specific ligand that can bind to a specific receptor, NPs can provide an active targeting effect such that the drug contained in the NPs shows improved targeting site efficiency (Watcharin et al., 2015). Based on the role of ASGPR in targeting therapy for liver cancer (Liu et al., 2014; Craparo et al., 2015), a free carboxyl group was linked to one end of P188 to synthesize P188 succinate (SP188) *via* an esterification reaction (Mei & Zeng, XXXX), and galactosyl-succinyl-poloxamer 188 (Gal-SP188) was then synthesized (Peça et al., 2012). Simultaneously, galactosyl-succinyl-poloxamer 188-polylactide-*co*-glycolide (Gal-SP188–PLGA) was synthesized with galactose, P188, and PLGA, and RBG-loaded Gal-SP188–PLGA nanoparticles (RGPPNs) were also prepared using the new material. Galactosyl residue could specifically bind to ASGPR, and thus more drug was internalized into liver cells to achieve active liver-targeting and an improved anti-tumor effect. The *in vitro* cellular uptake, cytotoxicity, and cellular apoptosis of the human liver cancer cell line, HepG2, were investigated to determine the inhibitory effect of NPs. The *in vivo* therapeutic effects of drug-loaded NPs were evaluated in the primary hepatocarcinogenic mouse model induced by diethylnitrosamine (DEN). The process of targeting cell internalization of nanoparticles prepared with galactosyl-modified polymers is schematically illustrated in Scheme S1 in the Supplementary data.

## 2. Materials and methods

### 2.1. Materials

RBG was purchased from Shanghai source leaf Biological Technology Co., Ltd. (Shanghai, China). P188 (MW: 8400), PLGA (50:50, MW: 40 kD), D-α-galactose (Gal), coumarin-6 (C6, purity 99%), D-α-tocopherol polyethylene glycol 1000 succinate (TPGS), sodium dodecyl sulfate (SDS, purity 99%), succinic anhydride, sulphonethane, dicyclohexylcarbodiimide (DCC), 4-dimethylaminopyridine (DMAP), DEN, tetrahydrofuran (THF), and polyethylene glycol 400 (PEG400) were purchased from Sigma-Aldrich (St. Louis, MO). Methanol and acetic acid were obtained from Tedia Company (HPLC grade, Fairfleld, OH). The 4-[3-(4-iodophenyl)-2-(4-nitrophenyl)-2*H*-5-tetrazolio]-1,3-benzene-sulfonate (WST-1) was obtained from Roche Applied Science (Basel, Switzerland). The 4,6-diamidino-2-phenylindole dihydrochloride (DAPI) was purchased from Fluka (Buche, Switzerland). All other chemicals and reagents of the highest quality were commercially available. HepG2 cells were offered by Chinese Academy of Medical Sciences Cell Center.

### 2.2. Synthesis and characterization of P188–PLGA, Gal-SP188, and Gal-SP188–PLGA

**Scheme 1.**
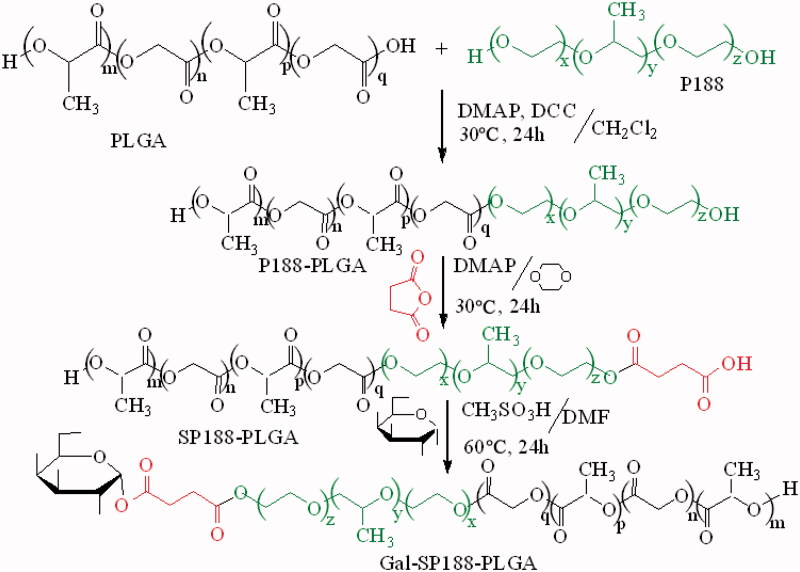
Illustration of the synthesis of Gal-SP188–PLGA polymer.

Briefly, succinyl-P188 (SP188) was synthesized *via* an esterification reaction. P188 (0.035 mmol) and succinic anhydride (0.04 mmol) were dissolved in dioxane (5 mL), followed by the addition of DMAP (0.1 mmol). This mixture was reacted at 30 °C for 24 h with stirring, and then it was precipitated with ethyl ether, filtrated and washed some times to remove unreacted reactants as complete as possible. Subsequently, the precipitation was dried to yield SP188 as a white powder. SP188 (0.012 mmol), sulphonethane (0.12 mmol), and Gal (0.12 mmol) were dissolved in dimethylformamide (DMF, 5 mL) to synthesize Gal-SP188. The mixed liquor was reacted at 60 °C for 24 h, and ethyl ether was chosen as the precipitant. After filtration and some washes with ethyl ether, the precipitation was dried to obtain Gal-SP188 as a white powder. Gal-SP188–PLGA was also synthesized *via* an esterification reaction. Briefly, PLGA (0.005 mmol), P188 (0.005 mmol), DMAP (0.016 mmol), and DCC (0.01 mmol) were dissolved in dichloromethane (10 mL), and the mixture was reacted at 30 °C for 24 h. Purified water was used to precipitate the product, and then the precipitation was vacuum-dried for 24 h after filtration and some washes to obtain the white power P188–PLGA. Next, according to the above method, succinyl-P188–PLGA (SP188–PLGA) was synthesized, and purified water was also used as a precipitant. SP188–PLGA was subsequently reacted with Gal at 60 °C for 24 h using sulphonethane as a catalyst and DMF (5 mL) as a solvent, and the polymer was precipitated as described for P188–PLGA and vacuum-dried for 24 h to obtain Gal-SP188–PLGA polymer as a white power (shown in Scheme 1). The complete methods performed to confirm these polymers had been successfully synthesized using the Fourier transform infrared (FTIR), ^1^H nuclear magnetic resonance (^1^H NMR), gel permeation chromatography (GPC), and differential scanning calorimetry (DSC) are described in the Supplementary data.

### 2.3. Preparation and characterization of RBG-loaded NPs

RBG-loaded Gal-SP188–PLGA nanoparticles (RGPPNs) were prepared using an ultrasonic emulsification-solvent evaporation method. Briefly, a certain amount of RBG and 100 mg Gal-SP188–PLGA copolymer were dissolved in 8 mL ethyl acetate to obtain an organic phase, and then the organic phase was dropped into 120 mL of the 0.03% (w/v) TPGS-purified aqueous phase in an ice bath while being sonicated at 100 W for 4 min to form an emulsion. The emulsion was continuously stirred at 500 r/min for 12 h to remove ethyl acetate as complete as possible. The NPs were collected by centrifugation using a high-speed refrigerated centrifuge (Sorvall Evolution RC; Kendro Laboratory Products, Asheville, NC) at 20,000 r/min for 15 min and washed some times with deionized water to remove unloaded drug and materials. Finally, the NPs, which were dispersed in 5 mL of deionized water, were lyophilized for 24 h and stored at 4 °C for further analysis. Empty Gal-SP188–PLGA nanoparticles (EGPPNs) were prepared in the same way without RBG. PLGA (100 mg), P188–PLGA (physical mixture consisting of 10.0 mg P188 and 100 mg PLGA), and Gal-SP188 & PLGA (physical mixture consisting of 10.0 mg Gal-SP188 and 100 mg PLGA) were chosen as carrier materials to generate RBG-loaded PLGA nanoparticles (RPNs), RBG-loaded P188–PLGA nanoparticles (RPPNs), and RBG-loaded Gal-SP188/PLGA nanoparticles (RGP&PNs), respectively, using the same protocol. In addition, coumarin-6(C6)-loaded PLGA nanoparticles (CPNs), C6-loaded P188–PLGA nanoparticles (CPPNs), C6-loaded Gal-SP188/PLGA nanoparticles (CGP&PNs), and C6-loaded Gal-SP188–PLGA nanoparticles (CGPPNs) were prepared in almost the same way except the fluorescent marker coumarin-6 was used instead of RBG. The complete methods used for characterization of these NPs are described in the Supplementary data.

### 2.4. *In vitro* cellular uptake of NPs

HepG2 cells were grown in high-glucose Dulbecco’s Modified Eagle’s Medium (H-DMEM) (Thermo Fisher Scientific, Waltham, MA) supplemented with 10% fetal bovine serum (FBS; Thermo Fisher Scientific) and 1% antibiotics. The cells were maintained in 5% CO_2_ at 37 °C in an incubator for measurements of cellular uptake of CPNs, CPPNs, CGP&PNs, and CGPPNs. For the qualitative analyzes, the complete methods used for these analyzes are described in the Supplementary data.

For the quantitative analyzes, all HepG2 cells in each well were collected and dispersed into purified water after observation for 30 min, 60 min by CLSM, respectively. The complete methods used for these analyzes are described in the Supplementary data.

### 2.5. Mechanism for cellular uptake of NPs

HepG2 cells in the logarithmic phase were seeded into six-well plates at a density of 1 × 10^5^ cells/mL and incubated in 5% CO_2_ at 37 °C for 24 h. The culture medium was then removed, and the cells were divided into four groups: the CGPPN, CGP&PN, CPPN, and CPN groups. Galactose solution (final concentration of 40 μg/mL) was added to one well in each group at the same time that the sucrose (final concentration of 450 mmol/L), sodium azide (NaN_3_) (final concentration of 25 mmol/L), methyl-β-cyclodextrin (final concentration of 3 nmol/L), genistein (final concentration of 200 μmol/L), and cytochalasin B (final concentration of 10 μg/mL) solutions were added to five separate wells in each group. The complete methods used for these analyzes are described in the Supplementary data.

### 2.6. *In vitro* cytotoxicity assays

The *in vitro* cytotoxicity of the nanoparticles was assessed using WST-1 assays (Ngamwongsatit et al., 2008). First, HepG2 cells were incubated with 100 µL culture medium in 5% CO_2_ at 37 °C in 96-well plates at a density of 1 × 10^4^ viable cells per well. After incubation for 24 h, the culture medium was removed and the cells were incubated with the RPN, RPPN, RGP&PN, and RGPPN suspensions and RBG solutions (RS, 0.1% dimethyl sulphoxide culture medium as solvents) at 1.25, 2.5, 5.0, 10.0, and 20.0 μg/mL final equivalent RBG concentrations, with 5-fluorouracil solutions (FS, commercial 5-fluorouracil for injection) at 1.25, 2.5, 5.0, 10.0, and 20.0 μg/mL final equivalent fluorouracil concentrations, and with EGPPN suspensions at the same Gal-P188–PLGA concentrations (4.9, 9.8, 19.6, 39.2, and 78.4 μg/mL) for 24, 48 and 72 h, respectively. The *in vitro* cytotoxicity of these NPs was evaluated using the dialysis method shown in the Supplementary data.

### 2.7. *In vitro* cell apoptosis

Annexin V-fluorescein isothiocyanate (FITC)/propidium iodide (PI) double staining assay was chosen to quantitatively detect the apoptosis of HepG2 cells among the RBG-loaded nanoparticles. First, HepG2 cells were incubated in H-DMEM at 37 °C in 5% CO_2_ for 24 h. Next, the HepG2 cells were divided into eight groups and a positive control group was cultured in FS at a final concentration of 20 μg/mL. The RPN, RPPN, RGP&PN, and RGPPN suspensions and RS were added (final concentration of RBG of 20 μg/mL), and EGPPN suspensions at the same Gal-P188–PLGA concentrations of 78.4 μg/mL were also added. Terminal deoxynucleotidyl transferase-mediated deoxyuridine triphosphate (dUTP) nick end labeling (TUNEL) assays were also performed to study the apoptosis of HepG2 cells qualitatively among the RBG-loaded NPs. The complete methods used for these analyzes are described in the Supplementary data.

### 2.8. *In vivo* therapeutic effects

#### 2.8.1. Establishment of the PLC mouse model

Kunming mice (Specific Pathogen Free grade) were provided by Dalian Medical University Laboratory Animal Centre (Dalian, People’s Republic of China). All the animal experiments in this study were approved by the Institutional Animal Care and Use Committee (IACUC) (Dalian Medical University, Dalian, China). The mice were all fed a standard laboratory diet and water before sacrifice and maintained under a constant temperature (22 ± 1 °C) and dark–light cycle (12 h/12 h). Ten mice were selected randomly as a normal control group without any drug administration. Ninety mice (6 weeks old, 20 ± 2 g) were randomly selected to establish the PLC mouse model, and the complete methods (Sengupta et al., 2014) are described in the Supplementary data.

#### 2.8.2. Drug administration and determination of biological samples

Two hundred microliters of 30% (v/v) PEG400-normal saline solutions were injected into each mouse in the normal control group. Sixty-four mice in the PLC model group were selected and divided randomly into eight groups (eight mice in each group). The complete methods used for drug administration (Chu et al., 2016) and determination of biological samples are described in the Supplementary data.

### 2.9. Statistical analysis

The results are presented as the mean ± sd. Statistical comparisons were performed using the Student’s *t*-test (SPSS13.0 statistical software) (International Business Machines Corporation, Armonk, NY), and a probability of *p* < .05 was considered statistically significant.

## 3. Results

### 3.1. Characterization of P188–PLGA, Gal-SP188, and Gal-SP188–PLGA

The FTIR spectra of the Gal-SP188–PLGA, Gal-SP188, P188–PLGA, PLGA, and P188 copolymers are shown in Figure S1. The major absorption bands for these polymers all had an O–H band in the region of 3500–3000 cm^−1^. However, the absorption of Gal-SP188 and Gal-SP188–PLGA was stronger than that of the other polymers because they had galactosyl groups with more O–H bands. In the FTIR spectra of the five polymers, the bands in the range of 2867–2949 cm^−1^ were assigned as –CH_2_ stretching bands. The carbonyl bands of PLGA, P188–PLGA, and Gal-SP188–PLGA with glycolide and lactide groups appeared at 1755 cm^−1^, but the absorption peak did not appear in P188 and Gal-SP188 because they did not have carbonyl bands. The absorption band at approximately 1091.7 cm^−1^ was attributed to C–O stretching. Therefore, the FTIR study confirmed that the Gal-SP188–PLGA diblock copolymer was successfully synthesized.

**Figure 1.**
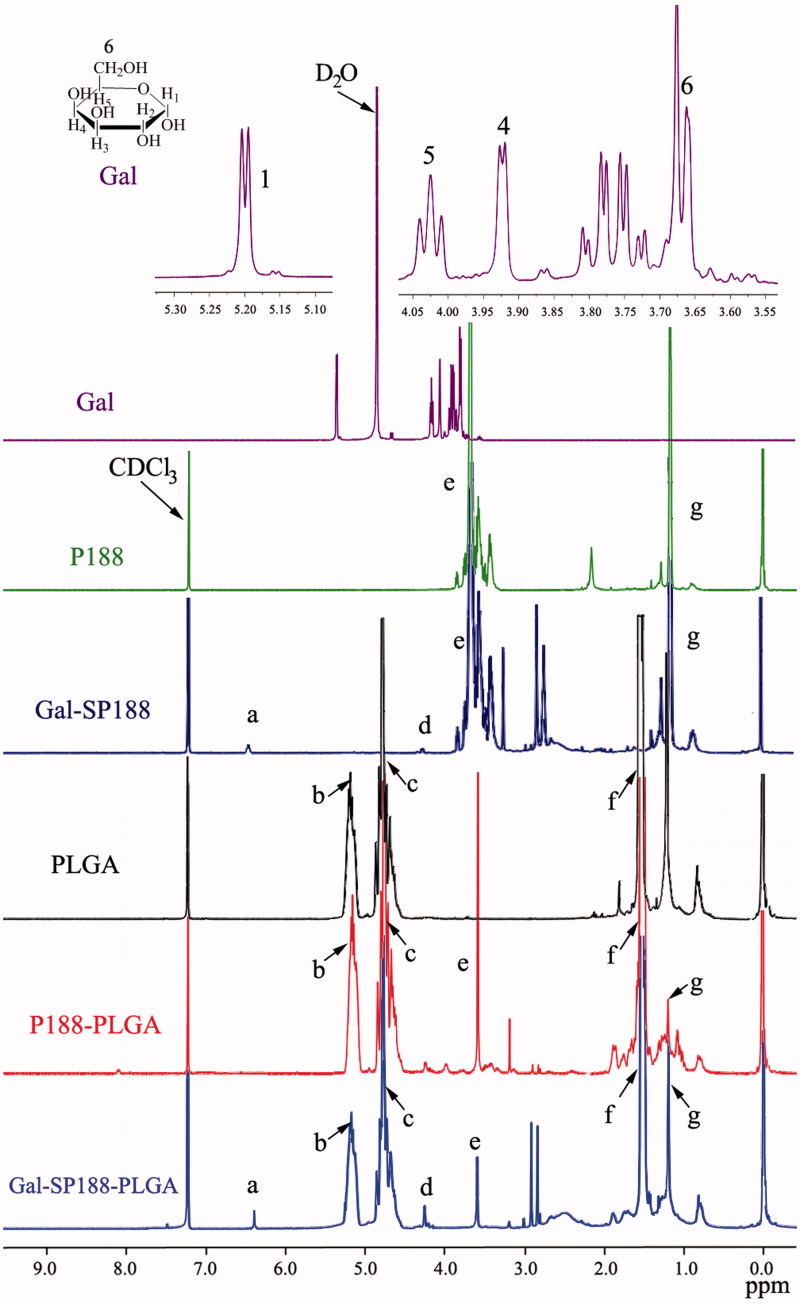
^1^H NMR spectra of Gal-SP188–PLGA, Gal-SP188, P188–PLGA, PLGA, and P188 copolymer.

The successful conjugation of Gal-SP188–PLGA was further confirmed by performing a ^1^H NMR study of all components (shown in Figure 1). The signals at 5.15 and 1.52 ppm (peaks b and f) were assigned to the CH and methyl (–CH_3_) protons of the PLA segment, respectively. The signals at 4.75 ppm (peaks c) were assigned to the CH_2_ protons of the PGA segment. The peaks at 3.58 ppm (peak e) and 1.18 ppm (peak g) were assigned to the –CH_2_ protons of the PEO part and the –CH_3_ protons of the PPO part of P188, respectively. The peaks at 6.43 (peak a) and 4.25 (peak d) were assigned to C_1_–H and other CH protons linked to hydroxyl groups of Gal, respectively. The ^1^H NMR of the P188, PLGA, P188–PLGA, and Gal-SP188 copolymers also exhibited peaks in similar regions because they had PLGA or P188 groups, respectively. The molecular weight of Gal-SP188–PLGA was calculated using the ratio between the peak areas at 5.15 (peak area 13.4), 4.75 (peak area 14.0), 3.58 (peak area 5.3), and 1.18 (peak area 1.2). The number-averaged molecular weight (Mn) of the Gal-SP188–PLGA random copolymer was determined to be 20,976. The ratios of PLGA and P188 molecular mass, which were integrated into the Gal-SP188–PLGA copolymer, were 84.71%, and 14.44%, respectively. The complete results of GPC (as shown in Figure S2) and DSC (as shown in Figure S3) analyzes for these polymers are described in the Supplementary data, respectively.

### 3.2. Characterization of RBG-loaded NPs

The size, size distribution, and ζ-potential of the NPs are listed in Table S1. The average particle size of the RGP&PNs and RGPPNs was within the range of 100–200 nm, which was smaller than that of the RPNs and RPPNs prepared with PLGA and P188–PLGA, respectively. As shown in Figure S4, the size distribution of RGP&PNs and RGPPNs was relatively narrow compared with the RPNs and RPPNs. All the NPs were negatively charged, and the ζ-potential was between −30 and −15 mV. There were no obvious differences between C6-loaded nanoparticles and RBG-loaded nanoparticles and the corresponding materials in terms of size, size distribution, and ζ-potential (as listed in Table S1).

**Figure 2.**
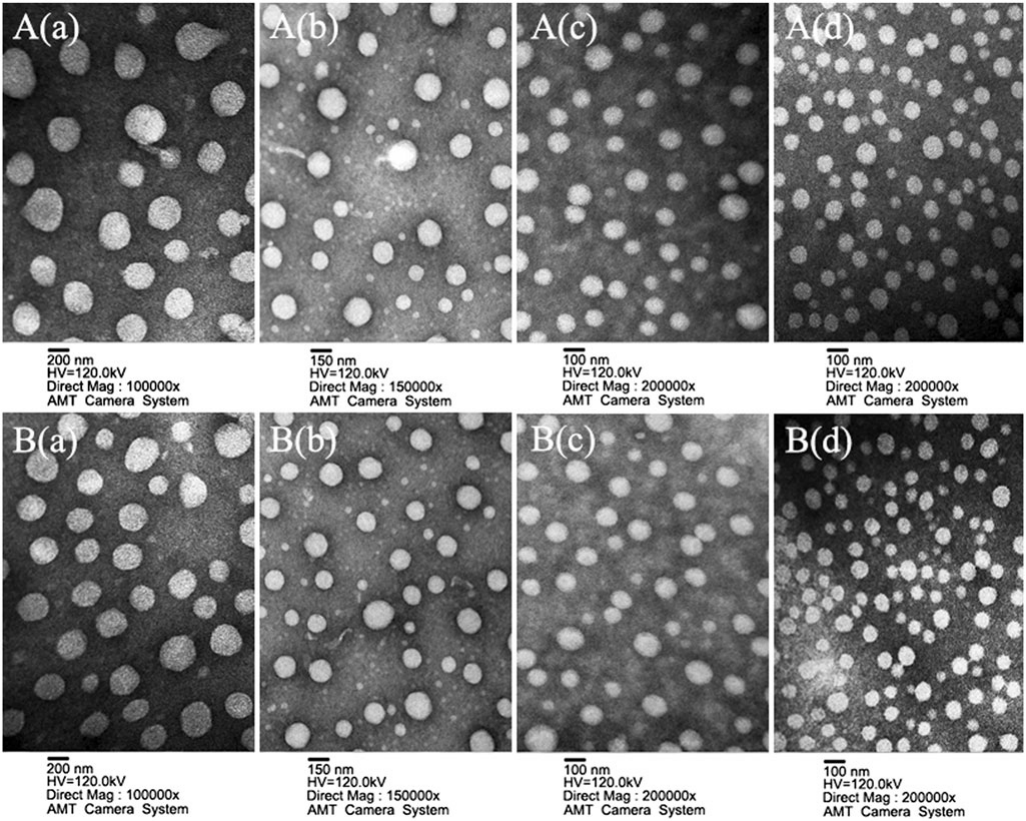
Characterization images of drug-loaded nanoparticles. A(a), A(b), A(c), and A(d) are TEM images of RBG-loaded PLGA nanoparticles (RPNs), RBG-loaded P188–PLGA nanoparticles (RPPNs), RBG-loaded Gal-SP188/PLGA nanoparticles (RGP&PNs), and RBG-loaded Gal-SP188–PLGA nanoparticles (RGPPNs), respectively. B(a), B(b), B(c), and B(d) are TEM images of C6-loaded PLGA nanoparticles (CPNs), C6-loaded P188–PLGA nanoparticles (CPPNs), C6-loaded Gal-SP188/PLGA nanoparticles (CGP&PNs), and C6-loaded Gal-SP188–PLGA nanoparticles (CGPPNs), respectively.

The particle size and morphology of these NPs were further studied using transmission electron microscopy (TEM) and scanning electron microscopy (SEM). The results (as shown in Figures 2 and S5) revealed that all NPs were spherical and regular with a uniform and monodispersed particle size distribution. The average particle size of the RPNs, CPNs, and RPPNs exceeded 200 nm, while that of the CPPNs, RGP&PNs, CGP&PNs, RGPPNs, and CGPPNs ranged from 100 to 200 nm, consistent with the results presented in Table S1. The complete results of DSC analysis (shown in Figure S6), drug loadings (DLs), encapsulation efficiency (EEs) and *in vitro* drug cumulative release profiles (shown in Table S1 and Figure S7) for these NPs are described in the Supplementary data.

### 3.3. *In vitro* cellular uptake of NPs

**Figure 3.**
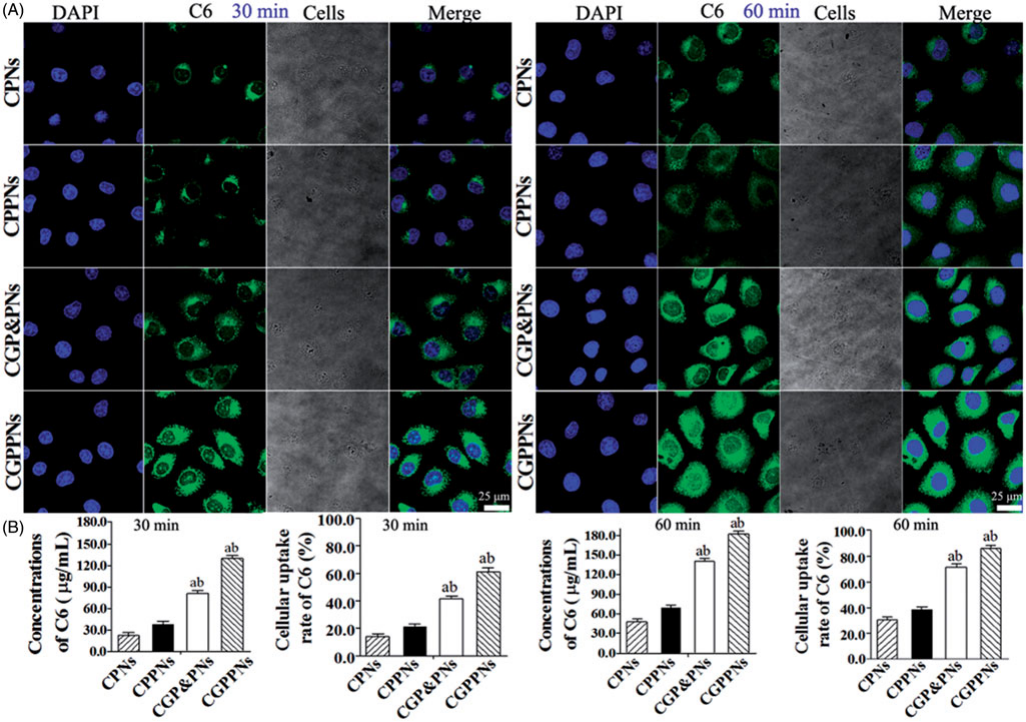
(A) The confocal laser scanning microscopy (CLSM) images of HepG2 cells after 30 and 60 min incubation with C6-loaded NPs suspensions, respectively. (B) The concentrations and cellular uptake rates of C6 in CPNs, CPPNs, CGP&PNs, and CGPPNs internalized by HepG2 cells after incubation for 30 and 60 min, respectively (*n* = 6, mean ± SD. ^a^*p* < .05 versus corresponding CPPNs; ^b^*p* < .01 versus corresponding CPNs).

The confocal laser scanning microscope (CLSM) images of HepG2 cells after incubation with suspensions of 200 μg/mL C6-loaded NPs for 30 min, 60 min at 37 °C in H-DMEM are displayed in Figure 3(A). All the nuclei of HepG2 cells stained with DAPI exhibited blue fluorescence. The intensity of green fluorescence increased significantly in HepG2 cells incubated with CGPPNs in comparison to those incubated with CPNs, CPPNs, and CGP&PNs. The concentrations and the cellular uptake rates of C6 in CPNs, CPPNs, CGP&PNs, and CGPPNs internalized by HepG2 cells after 30 min and 60 min are also shown in Figure 3(B), respectively. The results indicated that the cellular uptake rate of C6 in HepG2 cells incubated with CGP&PNs and CGPPNs increased by 40.6% and 55.1% (*p* < .01) and by 32.9% and 47.4% (*p* < .05) compared with CPNs and CPPNs at 60 min, respectively.

### 3.4. Mechanism for cellular uptake of NPs

As shown in Figure S8, CGPPNs and CPNs could be internalized by HepG2 cells. The cellular uptake rate of cells with the addition of only CPN and CPPN suspensions was decreased by 59.5%, 30.2% compared with the addition of CGPPN suspensions, respectively. Gal solutions had no significant impact on the cellular uptake rate of CPNs and CPPNs. Additionally, compared with the positive control group, the rate of cellular uptake of CGPPNs and CGP&PNs decreased significantly (by 40.2% and 29.9%, *p* < .01) following the addition of Gal solutions (shown in Figure S9), which suggested that the cellular uptake of CGPPNs and CGP&PNs was an active endocytic process mediated by ASGPR. When NaN_3_ solution was added, the cellular uptake rates of CGPPNs, CGP&PNs, CPPNs, and CPNs only decreased by 4.3%, 4.2%, 5.4%, and 5.6%, which suggested that the cellular uptake of these NPs was not an energy-dependent process. Hypertonic sucrose could inhibit the cellular uptake of these NPs, and the rates of cellular uptake of CGPPNs, CGP&PNs, CPPNs, and CPNs decreased by 27.5%, 19.4%, 46.5%, and 20.8%, respectively, which illustrated that the endocytosis of CPPNs was primarily mediated by clathrin. Following the addition of methyl-β-cyclodextrin, the cellular uptake rates of CGPPNs, CGP&PNs, CPPNs, and CPNs decreased by 15.4%, 15.7%, 9.3%, and 4.7%, respectively. In contrast, the genistein and cytochalasin B solutions had no significant effect on the cellular uptake of these NPs.

### 3.5. *In vitro* cytotoxicity assays

The *in vitro* cytotoxicities of RPNs, RPPNs, RGP&PNs, RGPPNs, RS, FS, and EGPPNs against HepG2 cells were determined using the WST-1 assay, and the results are shown in Figure S10. There were no significant changes in the cell viability ratio (CVR) for EGPPN in the presence of various Gal-SP188–PLGA concentrations, and the results were not statistically significant (*p* > .05) compared with the negative control group. Thus, the Gal-SP188–PLGA copolymer, which was newly synthesized to prepare nanoparticles in the present study, was considered to be biocompatible and nontoxic to cells. The complete results of *in vitro* cytotoxicity assays and half inhibitory concentration (IC_50_) values for these NPs (as listed in Table S2) are described in the Supplementary data.

### 3.6. *In vitro* cell apoptosis

As listed in Table S3, compared with the negative control group, the proportion of cells undergoing apoptosis increased significantly in the RBG-loaded NP group with extension of the incubation time. Compared with the FS group, the apoptosis ratio increased significantly (a difference of 44.7% and 62.4%, *p* < .01) in the RGPPN group at 24 and 72 h (also shown in Figure S11). The proportion of apoptotic cells was also increased by 5.3% and 5.5% (*p* > .05) in the RGPPN group at 24 and 72 h compared with the RGP&PN group, respectively. The percentage of apoptotic cells in each group increased with a greater incubation time, and the apoptosis ratio for RGPPNs and RGP&PNs at 24 and 72 h increased by 38.1% (*p* < .01) and 57.7% (*p* < .01) and by 32.8% (*p* < .01) and 52.2% (*p* < .01) compared with the RS group, respectively. After different culture times, the apoptosis ratio and the total level of cell death was highest for the RGPPN group compared with the other groups, consistent with the *in vitro* cytotoxicity results.

**Figure 4.**
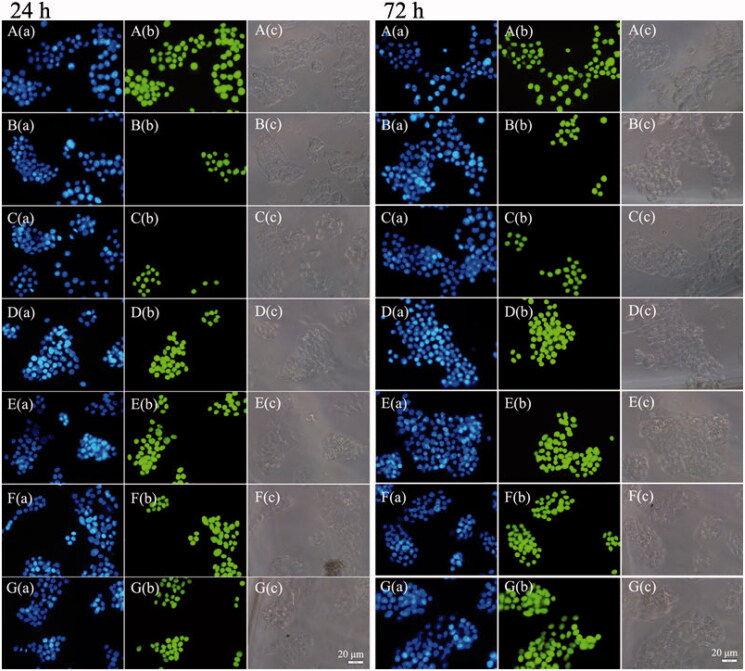
The fluorescence inversion microscopy (FIM) images of apoptotic HepG2 cells induced by FS, RS, RPNs, RPPNs, RGP&PNs, and RGPPNs after incubation for 24 and 72 h using TUNEL assays. Only apoptotic cellular nuclei displayed green fluorescence while all the nuclei were blue stained by DAPI. A(a)–G(a) are blue fluorescence field images of HepG2 cells in positive control, FS, RS, RPNs, RPPNs, RGP&PNs, and RGPPNs, respectively; A(b)–G(b) are green fluorescence field images of HepG2 cells nuclei disposed with TUNEL assays in positive control, FS, RS, RPNs, RPPNs, RGP&PNs, and RGPPNs, respectively; A(c)–G(c) are bright field images of HepG2 cells in positive control, FS, RS, RPNs, RPPNs, RGP&PNs, and RGPPNs, respectively.

The TUNEL assay results are shown in Figure 4. All the nuclei were stained with DAPI and exhibited blue fluorescence in all groups. All the nuclei in positive control group (Figure 4) exhibited green fluorescence, and only apoptotic cellular nuclei displayed green fluorescence in the other groups. The RGPPN group (Figure 4(G)) showed the highest amount of nuclear green fluorescence, with comparatively reduced levels in the RS group (Figure 4(C)) and the lowest levels in the FS group (Figure 4(B)). The bright field images demonstrated that the apoptotic cell bodies with green fluorescence decreased while their membranes became incomplete. The percentage of apoptotic cells in each group also increased with a greater incubation time, and the apoptosis ratio for RGPPNs and RGP&PNs at 24 and 72 h increased compared with the RS group, respectively. The apoptotic results in human hepatic sinusoidal endothelial cells (HHSECs) disposed with TUNEL assay decreased significantly in comparison to HepG2 cells (shown in Figure S12). The RGPPN and RGP&PN groups showed significantly less amount of nuclear green fluorescence at 72 h compared with them in HepG2 cells, respectively.

### 3.7. *In vivo* therapeutic effects

**Figure 5.**
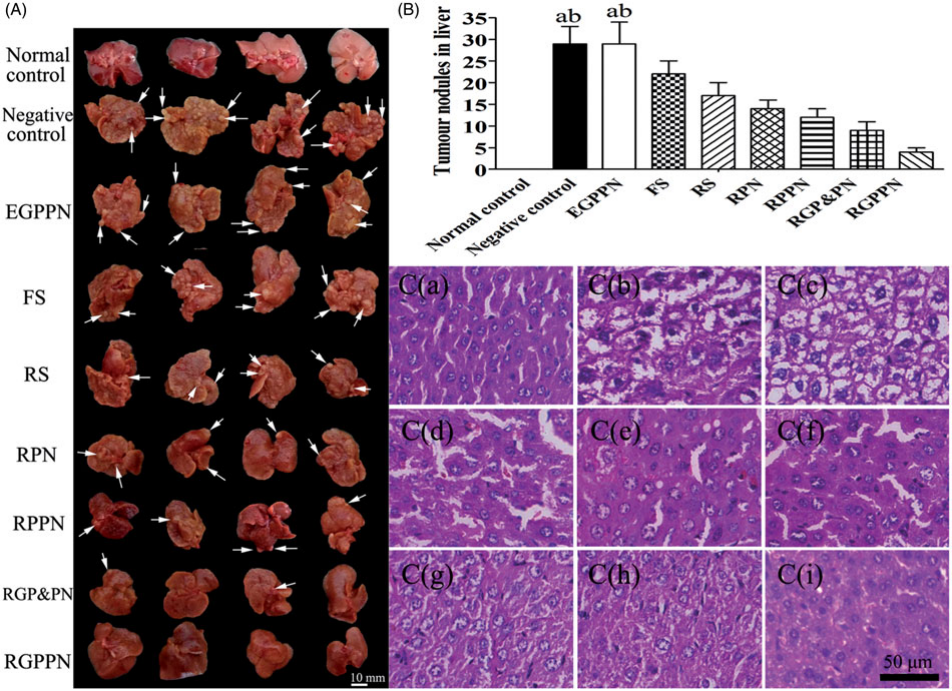
(A) Representative images of excised livers from the normal control and PLC model mice treated with 20% (v/v) PEG400–normal saline solution (negative control), EGPPN, FS, RS, RPN, RPPN, RGP&PN, RGPPN groups, respectively. (B) Tumor nodules in liver of PLC model mice treated with 20% (v/v) PEG400–normal saline solution (negative control), EGPPN, FS, RS, RPN, RPPN, RGP&PN, RGPPN groups after 30 days (*n* = 8, mean ± SD. ^a^*p* < .05 versus corresponding RGP&PN group; ^b^*p* < .01 versus corresponding RGPPN group). (C) The representative liver slices of each group stained with H&E and observed using microscope (×400). C(a)–C(i) are H&E images of the normal control, negative control, EGPPN, FS, RS, RPN, RPPN, RGP&PN, RGPPN groups, respectively.

Twelve mice with major pathological manifestations, such as severe cell degeneration, necrosis and lymphocytic infiltration, died during the 20-week modeling process; the mortality ratio was 13.33%. The indexes for the serum and liver homogenates of the remaining mice after administration and sacrifice met the requirements of the PLC model, and the success ratio was 86.66%. Figure 5(A) presents the morphology of parts of the livers of each group and Figure 5(B) presents the tumor nodules in liver of each group. The livers of each mouse except those of the normal control group showed hard, gray nodules varying in size from 3 to 10 mm. As listed in Table 1, compared with the normal control group, the contents of ALT, AST, γ-GT, and T-BIL in the serum of the negative control group increased by 65.5 U/L, 95.9 U/L, 12.8 U/L, and 10.2 μmol/L, respectively (*p* < .01), and the contents of ALT, AST, and γ-GT in liver homogenate increased by 12.2, 18.4, and 1.4 U/gprot (*p* < .01). The contents of ALB (in serum) and TP (in liver homogenate) decreased by 19.7, 7.8 g/L, respectively (*p* < .01), which suggested that the livers of the model mice suffered serious injuries that resulted in a decline in liver synthesis functions. There were no evident difference in the indexes between the negative control group and the EGPPN group (*p* > .05). Compared with the RPN group, the contents of ALT, AST, γ-GT, and T-BIL in the serum of the RGPPN group decreased by 33.3 U/L, 37.5 U/L, 5.3 U/L, and 3.3 μmol/L, respectively (*p* < .05). Compared with the FS group, the contents of ALT, AST, and γ-GT in the serum of the RGPPN group decreased by 28.2, 47.9, and 6.2 U/L (*p* < .05), the content of ALB in the serum of RGPPN group increased by 7.8 g/L (*p* < .01), and the content of T-BIL decreased by 4.4 μmol/L (*p* < .01). In addition, during the administration, a lower body weight was observed in the negative control group due to liver damage and a decrease in synthetic and metabolic liver functions. However, the body weight gain ratio in the RGPPN group increased rapidly because RGPPN had a certain therapeutic effect on mouse liver, as also shown in Figure S13.

**Table 1.** Liver functional indexes in the serum and liver of the primary hepatocarcinogenic mice (*n* = 8, mean ± SD).

		Liver function indexes in the serum					Liver function indexes in the liver			
Group	Liver index (g/kg)	ALT (U/L)	AST (U/L)	γ-GT (U/L)	T-BIL (μmol/L)	ALB (g/L)	ALT (U/gprot)	AST (U/gprot)	γ-GT (U/gprot)	TP (g/L)
Normal control	41.8 ± 1.3	30.7 ± 1.6	95.6 ± 1.4	3.3 ± 1.4	2.5 ± 1.4	39.4 ± 1.7	8.3 ± 1.4	14.1 ± 1.2	0.3 ± 0.1	16.7 ± 2.7
Negative control	58.9 ± 2.8^b^^d^	96.2 ± 2.9^b^^d^	191.5 ± 2.3^b^^d^	16.1 ± 2.8^b^^d^	12.7 ± 2.8^b^^d^	19.7 ± 2.7^b^^d^	20.5 ± 2.7^b^^d^	32.5 ± 2.5^b^^d^	1.7 ± 0.4^b^^d^	8.9 ± 1.4^b^^d^
EGPPN	57.7 ± 2.7^b^^d^	95.9 ± 2.9^b^^d^	190.8 ± 2.4^b^^d^	15.9 ± 2.7^b^^d^	12.1 ± 2.7^b^^d^	20.2 ± 2.5^b^^d^	20.9 ± 2.8^b^^d^	32.7 ± 2.7^b^^d^	1.8 ± 0.5^b^^d^	9.1 ± 1.5^b^^d^
FS	54.8 ± 2.4^a^^d^	77.5 ± 2.8^a^^c^	176.3 ± 2.1^a^^d^	11.5 ± 2.3^a^^d^	9.5 ± 2.9^b^^d^	22.9 ± 2.2^b^^d^	15.6 ± 2.5^b^^d^	25.4 ± 2.0^a^^d^	1.5 ± 0.4^b^^d^	10.5 ± 1.3^b^^d^
RS	55.6 ± 2.2^a^^d^	85.1 ± 2.7^a^^c^	181.1 ± 2.0^a^^c^	12.7 ± 2.2^a^^d^	9.8 ± 2.5^a^^d^	20.6 ± 1.9^a^^c^^e^	16.5 ± 2.1^a^^c^	24.1 ± 1.9^a^^d^	1.4 ± 0.5^b^^d^	10.4 ± 1.4^b^^d^
RPN	52.1 ± 2.0^a^^d^	82.6 ± 2.6^a^^c^	165.9 ± 2.2^a^^c^	10.6 ± 2.0^a^^d^	8.4 ± 2.3^a^^d^^e^	23.4 ± 2.1^a^^c^	14.6 ± 2.0^a^^c^	23.4 ± 2.1^a^^d^	1.3 ± 0.3^a^^c^	11.2 ± 1.7^a^^c^
RPPN	50.7 ± 2.3^a^^d^	78.4 ± 2.5^a^^d^	144.7 ± 1.9^a^^d^	8.4 ± 2.1^a^^d^	7.9 ± 2.2^a^^d^^e^	25.1 ± 1.9^a^^d^	13.4 ± 2.3^a^^d^	22.8 ± 2.4^b^^d^	1.1 ± 0.3^a^^c^	12.6 ± 1.9^a^^c^
RGP&PN	48.2 ± 1.9^e^	53.5 ± 2.1	130.2 ± 1.8	6.2 ± 1.9^c^^e^	6.6 ± 2.0^c^^f^	27.6 ± 1.5^f^	11.2 ± 1.8^e^	21.2 ± 1.8^d^^e^	0.9 ± 0.2^e^	13.5 ± 2.4^f^
RGPPN	46.5 ± 1.4	49.3 ± 1.8	128.4 ± 1.4	5.3 ± 1.5	5.1 ± 1.6	30.7 ± 1.4	10.8 ± 1.5	20.6 ± 1.4	0.5 ± 0.1	14.8 ± 2.8

Liver index (g/kg) = liver weight of mouse (g)/body weight of mouse (kg).

^a^ *p* < 0.05,

^b^ *p* < 0.01 versus corresponding RGPPN group;

^c^ *p* < 0.05,

^d^ *p* < 0.01 versus corresponding normal control group;

^e^ *p* < 0.05,

^f^ *p* < 0.01 versus corresponding negative control group.

The hematoxylin and eosin (H&E) staining results are shown in Figure 5(C). As shown in Figure 5(C(a)), in the normal control group, structural integrity of the lobules could be observed, the liver cell cord was arranged in neat rows, and the nuclei were clear without atypia. According to the Edmondson pathology classification in 1954 (34), Grade I cancer cells show mild abnormities and are arranged irregularly with obvious nuclei and numerous vacuoles in the cytoplasm; Grade II cancer cells show an increased level of atypia and close clumping, with larger nuclei and different shades of some vacuoles in the cytoplasm; Grade III cancer cells display obvious atypia with prominent nucleoli and irregularly arranged nuclei; and Grade IV cancer cells show clear morphological variations with large and irregular nuclei, a reduced amount of cytoplasm, and a loose arrangement of the cells. As shown in Figure 5(C(b) and (c)), there were no evident differences between the negative control group and the EGPPN group, obvious atypia was observed in the cancer cells and the shape of cells was highly irregular. In both groups, Grade IV was observed more frequently. As shown in Figure 5(C(d)), the FS group more frequently exhibited Grade III. As shown in Figure 5(C(e,g)), the RS, RPN, and RPPN groups more frequently showed Grade II, and the RGP&PN group (Figure 5(C(h))) was more frequently Grade II, while the RGPPN group (Figure 5(C(i))) was more frequently Grade I. Compared with the FS, RS, RPN, and RPPN groups, the levels of cell abnormity in the RGPPN group clearly decreased with obvious nuclei, which suggested that the therapeutic effect of the RGPPN group was superior to those of the FS, RS, RPN, and RPPN groups.

## 4. Discussion

For the first time, the Gal-SP188–PLGA, Gal-SP188, P188–PLGA random copolymers were successfully synthesized as nanoparticle carriers of small molecular anticancer drugs with RBG as a model drug.

In this study, RGPPN was prepared for the first time using the homemade materials Gal-SP188–PLGA as a carrier. Because RBG was loaded onto attachment materials of 100–200 nm, which have an improved passive targeting effect (Fox et al., 2009), RBG could be released slowly at the targeting site and thus reduce the toxicity of RBG. The P188 residues in RGPPN could influence the multi-drug resistance (MDR) of tumor cells and increased the tumor cell sensitivity to RBG (Kabanov et al., 2002). Concurrently, the Gal residues in RGPPN could combine specifically with ASGPR in liver (Ishibashi et al., 1994; Rigopoulou et al., 2012) such that RGPPN could have an active targeting effect to the liver and play a more effective therapeutic role for liver cancer.

The targeting effect of NPs was closely related to the particle size and surface charge, and the ζ-potential had an important impact on the stability of NP suspensions and the *in vivo* interaction with the cell membrane (Yan et al., 2010). To evaluate the quality of the NPs, the particle size, ζ-potential, DL, and EE of the RPNs, RPPNs, RGP&PNs, and RGPPNs were measured. The results showed that the average particle size of RGPPNs was significantly smaller than that of RPNs (*p* < .05), and the absolute value of the ζ-potential of RGPPNs was obviously higher than that of RPNs (*p* < .01); DL and EE of RGPPNs were the highest among these NPs. This finding might be explained by P188 as a kind of nonionic surfactant that is soluble in both water and organic solvent (Watcharin et al., 2015). P188 could also be used as a co-emulsifier in a nanometer system to obtain smaller particle sizes, and at the same time, its steric stabilization could prevent the aggregation of the already formed NPs and strengthen the hydrophilicity of PLGA. Thus, the content and solubility of RBG in RGPPNs were improved, DL and EE of RGPPNs were highest and the particle size of RGPPNs was smallest among the four kinds of RBG-loaded NPs.

The *in vitro* drug release of NPs was closely related to the *in vivo* pharmacodynamics. To maintain the sinking condition, 0.5% SDS was added as a solubilizer into PBS with isothermal medium supplementation. As shown in Figure S7, the four kinds of RBG-loaded NPs showed sustained-release effects and typical biphasic release compared with RS. During the initial burst phase in the first 5 days and the sustained release phase after 5 days, the RBG in the released NPs was nearly complete after 30 days. As a component of the nanomaterials, the P188 in RPPNs and Gal-SP188 in RGP&PNs and RGPPNs could not only provide the NPs with a hydrophilic environment, but they could also be used as the pore-forming agent to accelerate the degradation of the material and the release of RBG-loaded NPs. For the above reasons, the cumulative release rates of RPPNs, RGP&PNs, and RGPPNs were higher than those of RPN at the same time points. Compared with P188, the number of hydroxyl (–OH) groups increased in Gal-SP188 molecules. Gal-SP188 could form more intermolecular hydrogen bonds with H_2_O molecules in the release medium, thereby speeding up the degradation of the materials and the release of RBG. Therefore, the cumulative release rates of RGP&PNs and RGPPNs were higher than that of RPPNs. Additionally, compared with RGP&PNs, RGPPNs had a smaller particle size and a narrower distribution, which resulted in a larger area in contact with the release medium. The content of Gal-SP188 in RGPPNs was greater than that of Gal-SP188/PLGA in this study, which could make RGPPNs more hydrophilic and Gal-SP188–PLGA degrade faster in the release medium. Thus, the *in vitro* cumulative release rate of RBG in RGPPNs, which was highest after 30 days (>90%), was faster and more complete.

In this research, CPNs, CPPNs, CGP&PNs, and CGPPNs were prepared to discuss the cellular uptake efficiency by HepG2 cells, which greatly influenced the therapeutic effect of the drug-loaded NPs. RBG did not exhibit fluorescence in cells, and C6 was used as a fluorescent probe in the nanoparticle formulations to visualize and analyze the cellular uptake of NPs for its perfect biocompatibility, high fluorescence ability, and a low leakage rate (Panyama et al., 2003). The cellular uptake efficiency of CGPPNs was much higher than that of CPNs and CPPNs at different time points, further confirming that the use of Gal-P188–PLGA as the material for drug-loaded NPs could achieve a higher cellular uptake efficiency through ligand–receptor binding in HepG2 cells.

HepG2 cells were chosen to study the cellular uptake mechanism of CGPPNs, CGP&PNs, CPPNs, and CPNs, and the cellular uptake rates of these NPs after the addition of different inhibitors were also investigated. The cellular uptake rates of these NPs were determined when Gal solutions were added as a competitive inhibitor. The results showed that Gal had no obvious influence on the cellular uptake rates of CPPNs and CPNs because there were no Gal residues in CPPNs and CPNs, and the Gal solutions did not show a competitive inhibition effect on CPPNs and CPNs through ASGPR. While CGPPNs and CGP&PNs containing Gal residues were influenced by the competitive inhibition of Gal solutions, the cellular uptake rates of CGPPNs and CGP&PNs decreased significantly (*p* < .01). These results indicated that CGPPNs and CGP&PNs had an active liver-targeting effect by recognizing and binding to ASGPR. Hypertonic sucrose, NaN_3_, methyl-β-cyclodextrin, genistein, and cytochalasin B were chosen as transport inhibitors to study the effect of the inhibitors on the cellular uptake rates of these NPs. Hypertonic sucrose could inhibit endocytosis mediated by clathrin and caveolin by concomitantly destroying the formation of caveolin. The results showed that sucrose could inhibit the uptake of CGPPNs and CGP&PNs of HepG2 cells, which suggested that the cellular uptake mechanism of CGPPNs and CGP&PNs was associated with clathrin and caveolin. The cellular uptake rates of these NPs less decreased when NaN_3_ solution was added as an energy inhibitor, which illustrated that the cellular uptake of these NPs was not energy-dependent. Genistein is a tyrosine kinase inhibitor that can inhibit endocytosis mediated by clathrin. Endocytosis is a common mechanism of exogenous nanoparticle uptake, and endocytosis mediated by clathrin is one of the main routes of NP uptake (Liu et al., 2004). Cytochalasin B is a kind of actin inhibitor (Zhu et al., 2016), and methyl-β-cyclodextrin is a water-soluble cyclic glucopyranose oligomer. When cytochalasin B or genistein was added to the samples, the cellular uptake of the NPs showed no obvious differences compared with the control group, which indicated that cytochalasin B and genistein had no significant effect on the cellular uptake of these NPs. In summary, the cellular uptake of CGPPNs and CGP&PNs by HepG2 cells occurred *via* active liver-targeting endocytosis mediated by ASGPR, and this process could be mediated by caveolin and clathrin.

In this study, HepG2 cells were used for the *in vitro* cytotoxicity of RPNs, RPPNs, RGP&PNs, and RGPPNs, and FS was chosen as a positive control drug to investigate the *in vitro* inhibitory effect of RBG-loaded NPs on liver cancer cells because 5-fluorouracil is a chemotherapeutic drug that is commonly used to treat transhepatic arterial chemotherapy and embolization (TACE). Furthermore, it can be injected *via* an intravenous route, which is coincident with the delivery method chosen for the NPs. The results showed that the newly synthesized Gal-SP188–PLGA material was biocompatible and non-cytotoxic. With an increasing concentration of RBG, the CVR in HepG2 cells decreased in, to some degree, a time-dependent and dose-dependent manner. The longer the incubation time, the lower was the CVR, and the RGPPN group exhibited higher toxic effects than the RPN, RPPN, and RGP&PN groups, which was consistent with the fluorescence intensity and RBG content in the *cellular uptake* test. Since Gal residues in the RGPPN and RGP&PN materials were capable of binding to ASGPR in the HepG2 cell membrane, more NPs were internalized into cells and the drug concentration in the cells increased (Cheng et al., 2012; Rigopoulou et al., 2012; Zhu et al., 2013). Regarding the RGPPN group, Gal-SP188 and PLGA were chemically bonded, and the P188 chemically modified material could enhance the hydrophily of NPs as well as reverse the drug resistance of tumor cells (Kabanov et al., 2002). Compared with the physical mixture of Gal-SP188 and PLGA, Gal-SP188–PLGA had more Gal residues on the surface of NPs and some advantages such as uniform material, good physical stability, and highly efficient cellular uptake. Thus, RGPPNs could play a superior role compared with RGP&PNs in the active targeting effect and inhibition of liver cancer cells. The fluorescence intensity in RGPPNs was also significantly stronger than that of RGP&PNs, RPPNs, and RPNs in the cellular uptake assays. The IC_50_ values in the RGPPN and RGP&PN groups were significantly lower than those in the FS and RS groups at the same times and concentrations. Compared with the RBG solutions, the NP formulation with the sustained release function and active targeting effect for specific receptors allowed more RBG-loaded NPs to enter cells and more completely release RBG. As a result, the NPs could prolong the effect of RBG in cells and achieve a better anti-cancer effect. The CVR for the RGPPN group at different time points and different concentrations (Figure S10) indicated that the optimal administration dose of RGPPNs was 20 μg/mL, and it laid the foundation for the *in vivo* pharmacodynamic study of RGPPNs.

Cell apoptosis is an initial cell death process throughout the entire life span of an organism and an important way to maintain homeostasis in the body (Huber et al., 2010). The rapid growth of liver cancer results from increased proliferation and reduced apoptosis of cancer cells, and drug-induced cancer cell apoptosis is a feasible method for cancer control and treatment (Choi et al., 2015). In the present study, FACS was used to detect cells stained with PI and Annexin V-FITC. Cells that were simply stained with PI were mechanically damaged, and those stained with Annexin V-FITC alone were early apoptotic cells; double-stained cells were secondary necrotic cells. For the apoptotic cells, the terminal 3′-OH was produced during DNA fragmentation and could be marked by dUTP, which was labeled with FITC, mediated by terminal deoxynucleotidyl transferase (TdT). When observed under a fluorescence microscope, apoptotic cell nuclei exhibited green fluorescence. As shown in Figure 4, the proportions of apoptosis in the RBG-loaded NP groups were higher than those in the RS and FS groups, which was consistent with the FACS analysis. The constant uptake of NPs by cells and the sustained drug release increased the intracellular accumulation of the drug to enhance the cell apoptosis effect. Because FS and RS had no sustained release properties, the apoptosis ratio of FS and RS at 72 h showed no obvious increase in comparison to 24 h. In the RGPPN and RGP&PN groups, Gal residues in the material were capable of being recognized specifically by ASGPR of HepG2 cells, and thus NPs could enter the cells by receptor-mediated endocytosis. Compared with the RPN, RPPN, and RS groups, the concentration of RBG in the RGPPN and RGP&PN groups further increased in HepG2 cells (Cheng et al., 2012; Rigopoulou et al., 2012; Zhu et al., 2013). The Gal-SP188 residues in the RGPPN group increased the hydrophilicity of NPs and reversed the resistance of cancer cells (Kabanov et al., 2002). Compared with the RGP&PN group, RGPPNs had some better characteristics, such as higher cellular uptake and more complete drug release, which was also confirmed by the *in vitro* cellular uptake and cell cytotoxicity assays in this study. Consequently, RGPPNs exhibited a stronger effect than RGP&PNs on apoptosis induction in HepG2 cells. Annexin V-FITC/PI double staining and TUNEL assays quantitatively and qualitatively indicated that RGPPNs prepared with the new synthetic Gal-SP188–PLGA had better active targeting functions and a stronger inhibitory effect on liver cancer cells with high ASGPR expression. The apoptotic ratio in HHSECs at 72 h disposed with TUNEL assay decreased significantly because no ASGPR was present in HHSECs, while ASGPR present in HepG2 cells could recognize and bind to the galactose residues (Roggenbuck et al., 2012) in RGPPNs.

In this study, the PLC mouse model was induced by DEN. DEN with nitrosamines had a strong toxicity towards the liver and is a denaturant and carcinogen. DEN-inducing liver cancer processes are similar to that of human PLC (Demirci et al., 2015). Compared with the xenograft tumor model, the PLC model showed good specificity and a higher success rate, and the liver cancer was mostly hepatocellular carcinoma. AST, ALT, TP, ALB, γ-GT, and T-BIL are clinically commonly used indicators of liver function assessments. Among them, AST and ALT mainly reflect the damage to liver cells, TP and ALB mainly reflect the synthetic function of liver, γ-GT is a positive marker of early variation and a characteristic index of precancerous lesions of liver cancer, and T-BIL mainly reflects the metabolic function of liver cells. In this study, the contents of AST and ALT in the serum in the negative control group increased significantly (*p* < .01) compared with the normal control group, suggesting that the liver cells in the negative control group were badly damaged. The contents of TP (*p* < .01) and ALB (*p* < .05) decreased in a non-significant manner, which indicated that the synthetic function of the liver in the negative control group declined. The content of T-BIL significantly increased (*p* < .01), indicating that the metabolic function of the liver in the negative control group seriously decreased. The content of γ-GT clearly increased (*p* < .01), prompting serious pathological changes in the liver, and reached the requirements for the PLC model. There were no palpable differences in the indexes in serum and liver homogenate between the EGPPN group and the negative control group, based on the results of H&E staining, validating the nontoxicity of the synthetic materials to normal tissues and cells. In our preliminary experiments, the mortality ratio of the mice was higher in the RS or FS group administered once per day because RS and FS had strong irritant effects and no selectivity. The livers were seriously injured in the mouse model, which caused the mice to become too weak to bear the administered dosage. In addition, RPNs, RPPNs, RGP&PNs, and RGPPNs maintained effectiveness for a long time because the NPs had a sustained-release effect. Thus, the administration at a certain time once every 3 days was chosen to reduce the irritant and toxicity properties of RS and FS, and the sustained-release effect of NPs was verified at the same time. Compared with the RS, RPN, and RPPN groups, the serum and liver homogenate indexes in the RGPPN group improved significantly (*p* < .01). Concomitantly, the H&E staining results also showed that the cell atypia was inconspicuous in the RGPPN group, which was frequently Grade I, compared with the RPN and RPPN groups. That result was because the Gal residues in the nanoparticle materials in the RGPPN group were capable of specifically recognizing ASGPR on the liver cell membrane, and thus the RGPPNs had good active liver-targeting properties and could facilitate the targeting of more RBG to liver (Cheng et al., 2012; Rigopoulou et al., 2012; Zhu et al., 2013) and further increase its liver cell uptake. Simultaneously, the Gal-SP188 group in RGPPNs enhanced the hydrophilic property of NPs and functioned to reverse the multi-drug resistance of cancer cells (Kabanov et al., 2002). Thus, the drug-loaded NPs could be released more rapidly and more completely (as shown in the *in vitro* drug release). Compared with the RPN and RPPN groups, the contents of γ-GT, ALT, and AST in the serum and liver homogenate in the RGPPN group decreased significantly (*p* < .05), the content of ALB in serum and TP in the liver homogenate increased (*p* < .05), and the content of T-BIL in serum decreased (*p* < .05). These results indicated that RGPPNs could better repair liver cell damage and improve the synthetic and metabolic function of the liver. In addition, during the administration, the body weight gain rate was lower in the negative control group due to the damage and decrease in the synthetic and metabolic function of the liver. However, the body weight gain rate in the RGPPN group increased more rapidly because RGPPNs have a certain therapeutic effect on the livers of PLC mice. In conclusion, the results indicated that the newly synthesized Gal-SP188–PLGA material had better cell and tissue compatibility both *in vivo* and *in vitro*, and RGPPN prepared with the material had superior anticancer effects *in vivo* and *in vitro* as well as strong active liver-targeting properties. The therapeutic effects of RGPPNs for PLC were significantly enhanced compared with RS and RPNs, and thus RGPPNs are expected to become a new option for the clinical treatment of PLC.

## 5. Conclusions

In this study, Gal-SP188–PLGA was synthesized for the first time as a novel material, and the FTIR, ^1^H NMR, GPC, and DSC were performed to confirm that the polymer had been successfully synthesized. Gal-SP188 and P188–PLGA were also synthesized simultaneously as controls. RPNs, RPPNs, RGP&PNs, and RGPPNs were successfully prepared using an ultrasonic emulsification–solvent evaporation method. All the NPs were spherical, negatively charged, and demonstrated a ζ-potential between −40 and −20 mV. The average particle size of the RPNs and RPPNs was greater than 200 nm, while that of RGP&PNs and RGPPNs ranged from 100 to 200 nm. All the NPs showed a typical biphasic release *in vitro*, and after 30 days, the cumulative release rates of RGP&PNs and RGPPNs were greater than those of RPNs and RPPNs because Gal-SP188 increased the hydrophilicity of RGP&PNs and RGPPNs and accelerated the drug release. The results of the cellular uptake assay showed that CGPPNs could be better internalized by HepG2 cells. The cellular uptake of CGPPNs and CGP&PNs by HepG2 cells occurred *via* active liver-targeting endocytosis mediated by ASGPR, and this process could be mediated by caveolin and clathrin. The WST-1 assays indicated that Gal-SP188–PLGA was nontoxic and biocompatible with HepG2 cells. More RBG could be internalized into cells because RGPPNs had an active targeting effect in liver cells, and thus RGPPNs showed higher cytotoxicity than RPNs, RPPNs, and RGP&PNs. The IC_50_ value of the RGPPNs decreased significantly in comparison to RS, FS, and RPNs, suggesting that RGPPNs could concentrate preferably in liver and concomitantly reduce the toxicity of RBG. Regarding apoptosis, RGPPNs exhibited a stronger effect on the induction of apoptosis in HepG2 cells than RPNs, RPPNs, and RGP&PNs, which indicated that RGPPNs prepared with the newly synthesized Gal-SP188–PLGA had better active targeting functions and a stronger inhibitory effect on liver cancer cells with high levels of ASGPR expression. The results obtained for the therapeutic effect *in vivo* illustrated that the indexes for serum and liver homogenates in the RGPPN group improved significantly; this group was frequently Grade I in the Edmondson pathology classification. Because RGPPNs could be internalized into liver cells through ASGPR, more RBG became concentrated in liver cells and exerted a better effect. Therefore, RGPPNs prepared with the newly synthesized Gal-SP188–PLGA biomaterial are expected to become a new and more effective treatment for PLC.
