# Correction to: Dong et al., Promising galactose-decorated biodegradable poloxamer 188-PLGA diblock copolymer nanoparticles of resibufogenin for enhancing liver cancer therapy

**DOI:** 10.1080/10717544.2017.1394511

**Published:** 2017-10-25

Dong H, Tian L, Gao M, Xu X, Zhang C, Lv L, Zhang J, Wang C, Tian Y and Ma X. (2017). Promising galactose-decorated biodegradable poloxamer 188-PLGA diblock copolymer nanoparticles of resibufogenin for enhancing liver cancer therapy. *Drug Delivery.* 24(1):1148–1158. https://doi.org/10.1080/10717544.2017.1373165.

When the above article was first published online, Figures 3 and 5 were incorrect.

This has now been corrected.

